# Change in health status (EQ-5D) over 5 years among individuals with and without type 2 diabetes mellitus in the SHIELD longitudinal study

**DOI:** 10.1186/1477-7525-10-99

**Published:** 2012-08-21

**Authors:** Susan Grandy, Kathleen M Fox

**Affiliations:** 1 AstraZeneca LP, Wilmington, DE, USA; 2 Strategic Healthcare Solutions, LLC, PO Box 543, Monkton, MD, 21111, USA

**Keywords:** EQ-5D, Type 2 diabetes mellitus, Quality of life, Longitudinal

## Abstract

**Background:**

Health-related quality of life studies among adults with type 2 diabetes mellitus, using the EQ-5D, have been short term and have not assessed change over years. This study assessed the change in health status and health-related quality of life over 5 years among individuals with and without diabetes.

**Methods:**

Respondents to the US **S**tudy to **H**elp **I**mprove **E**arly evaluation and management of risk factors **L**eading to **D**iabetes (SHIELD) completed the EuroQol-5D (EQ-5D) at baseline (2004) and 5 years later (2009). Visual analog scale (VAS) score and health index score were computed at baseline and year 5, and the change over 5 years was measured for individuals with type 2 diabetes mellitus (T2DM) and those without diabetes, and T2DM adults with and without diabetic complications. Linear regression models were used to determine change in EQ-5D score, controlling for age, gender, race, education, household income, and body mass index (BMI).

**Results:**

There was significantly greater decline in the EQ-5D index score in the T2DM group (-0.031 [SD 0.158]), compared with those without diabetes (-0.016 [0.141], p = 0.001). Compared with respondents without diabetes, those with T2DM had a larger reduction in EQ-5D index score, after controlling for demographics (p = 0.001). EQ-5D VAS score declined over 5 years for both groups: -1.42 (18.1) for the T2DM group, and -0.63 (15.8) for the group without diabetes, but the between-group difference was not significant either before (p = 0.09) or after (p = 0.12), controlling for demographics. T2DM respondents with diabetic complications had a greater decline in EQ-5D scores than T2DM respondents without complications (p < 0.05).

**Conclusion:**

Over a 5-year period, health status of respondents with T2DM declined significantly compared with those with no diabetes, indicating that the burden of the disease has a long-term detrimental impact. This decline in health status is likely to impact utility scores (fewer quality-adjusted life years) for economic evaluations.

## Background

During the past decade, there has been a dramatic rise in diabetes [1]. In the United States, there are 23.5 million adults 20 years or older with diabetes [2]. Worldwide, an estimated 366 million people had diabetes in 2011, with the number of people with type 2 diabetes mellitus (T2DM) increasing in every country [3]. Complications from diabetes include blindness, kidney disease, macrovascular disease, and neuropathy; diabetes is the seventh leading cause of death in the US [4].

Individuals with T2DM are known to have lower health-related quality of life (HRQOL) and more depressive symptomatology than those without diabetes [5-8]. Significant reductions in health status among diabetes patients compared with other chronic disease populations have been demonstrated using generic HRQOL instruments [5]. T2DM has been shown to be frequently associated with adverse psychological effects, particularly depression [5,6]. Among Michigan patients with T2DM, the number of diabetes complications was correlated with lower HRQOL scores [8]. In a population-based study of adults with and without T2DM, investigators found EQ-5D index scores and visual analog scores were significantly lower for respondents with T2DM and those with 3−5 risk factors for T2DM than for those with 0−2 risk factors [9]. The majority of these HRQOL studies in diabetes has focused on the current health state of patients and has not assessed change in health state over long periods of time, except for clinical trials that are short term.

HRQOL measures are used in the evaluation of healthcare interventions, including cost-effectiveness analyses [10]. For this purpose, quality-adjusted life years (QALYs) should be calculated using preferences from the population as a whole. Studies have suggested that patients’ values for their own health state affect their decisions concerning treatment and its health outcomes [11-13]. The EQ-5D is one HRQOL measure that has been calibrated with preferences from the whole population. Assessments of HRQOL using generic instruments like the EQ-5D allow for comparisons with other chronic diseases as well as comparisons with healthy populations to estimate the incremental burden of diabetes.

The objective of this present study was to assess the change in health status and HRQOL over a 5-year period among adults with and without T2DM, using data from the Study to Help Improve Early evaluation and management of risk factors Leading to Diabetes (SHIELD). Also, the change in health status and HRQOL among T2DM adults with diabetic complications was assessed to determine whether complications led to additional disease burden impact. Unlike clinical trials, this study evaluated change in HRQOL over 5 years among adults being treated in routine clinical practice. Until now, the EQ-5D has mostly been used in short-term studies of less than 2 years [14-16]. Evaluating change in EQ-5D scores over 5 years will elucidate whether health status and HRQOL would decline over a long period time, possibly indicating that longer duration with the burden of T2DM may lead to continued decline in HRQOL rather than adjusting to the disease and stabilization of HRQOL.

## Methods

This study is a longitudinal analysis of EQ-5D data collected in 2004 and 2009 among SHIELD respondents with T2DM and those with no diabetes. SHIELD is a 5-year, survey-based study conducted to better understand patterns of health status, health behavior, and knowledge and attitudes of people living with diabetes and those with varying levels of cardiometabolic risk.

### SHIELD survey

SHIELD included an initial screening phase to identify cases of interest in the general population (e.g., diabetes mellitus) and a baseline questionnaire to follow up identified cases and collect data regarding health status, health knowledge and attitudes, and current behaviors and treatments. Subsequently, annual follow-up surveys were administered for 5 years. A detailed description of the SHIELD methodology has been published previously [17,18].

In brief, the screening survey was mailed in April 2004 to a stratified random sample of 200,000 US households, representative of the US population for geographic residence, household size and income, and age of head of household [19], identified by the Taylor Nelson Sofres National Family Opinion (TNS NFO) panel (Greenwich, CT). All TNS NFO surveys were voluntary, and no special incentives were provided. A response rate of 64% was obtained for the screening survey. The SHIELD study was approved by the Quorum Review Board.

A comprehensive baseline survey was mailed in September–October 2004 to a representative sample of individuals 18 years of age or older (n = 22,001) who were identified in the screening survey as having self-reported type 1 diabetes mellitus or T2DM, no diabetes, or being at risk for diabetes. Each respondent group was balanced to be representative of that segment of the population for age, gender, geographic region, household size, and income for the US population, and then a random sample from each group was selected and sent the baseline survey. A response rate of 72% was obtained for the baseline survey. The 2009 annual follow-up survey (response rate of 70%) repeated the EQ-5D instrument for comparison with the baseline EQ-5D responses.

### Study measures

Respondents were classified as having T2DM based on their self-report of having been told by a doctor, nurse, or other healthcare professional that they had T2DM. Respondents who reported a diagnosis of type 1 diabetes mellitus or unspecified diabetes were excluded from the analysis. Respondents who did not report a diagnosis of T2DM, type 1 diabetes, or unspecified diabetes at baseline were included in the “no diabetes” group.

The EQ-5D was used as a measure of respondents’ HRQOL and utility values. The EQ-5D includes a descriptive profile and a single index value for health status [20,21]. The visual analog scale (VAS) records the respondents’ self-rating for their current HRQOL on a graduated (0−100) scale, with higher scores for higher HRQOL [22]. The descriptive system is composed of 5 dimensions of health: mobility, self-care, usual activities, pain/discomfort, and anxiety/depression. The VAS provides a direct valuation of the respondent’s current state of health, whereas the descriptive system can be converted into an index score representing a von Neumann-Morgenstern utility value for current health [20]. The health states from each of the 5 dimensions are converted into a weighted health state index by applying scores from the EQ-5D preference weights elicited from general population samples. These weights lie on a scale on which full health has a value of 1 and dead a value of 0. For this study, US population weights were used to convert to an EQ-5D index score [23].

Diabetes complications reported at the annual follow-up surveys included ever being diagnosed with retinopathy, neuropathy, and nephropathy. Respondents were classified as having retinopathy if they reported a diagnosis of eye disease, blindness, or retinopathy. Neuropathy was defined as reporting a diagnosis of nerve problems of the hands or feet involving pain, tingling, or numbness, foot ulcers, or amputation. Nephropathy was defined as a reported diagnosis of chronic kidney disease, dialysis, end-stage kidney disease, kidney transplant, or protein in the urine.

### Statistical analysis

EQ-5D VAS score and health index score were computed at baseline and 5 years later, and the change over 5 years measured for respondents with and without T2DM. Comparisons between respondents with and without reported T2DM and comparisons between T2DM respondents with and without diabetic complications were conducted using chi-square test for categorical variables and *t*-tests for continuous variables. Ordinary linear regression model was used to evaluate change in EQ-5D score by diabetes status (T2DM vs. no diabetes), controlling for age, gender, education, household income, and body mass index (BMI) as reported at baseline. Statistical significance was set *a priori* as p < 0.05.

## Results

There were 1,741 respondents with T2DM and 4,543 respondents without diabetes who completed the 2004 and 2009 EQ-5D questionnaires and were included in the analysis. T2DM respondents were significantly older (mean age: 60.6 vs. 56.1 years), had higher BMI (33.7 vs. 29.8 kg/m^2^), and a greater percentage had low education (35.1% vs. 28.5% with high school degree or less) and low household income (51.9% vs. 41.8% with annual income < $40,000), compared with respondents without diabetes (p < 0.001 for each comparison) (Table 1). At baseline (2004), 66.6% of the T2DM respondents received oral antidiabetic medications alone, 8.7% received insulin plus oral antidiabetic agents, 5.5% received insulin alone, and 19.2% received no diabetes medications. In 2009, 51.1% of the T2DM respondents received oral antidiabetic medications alone, 12.5% received insulin plus oral antidiabetic agents, 9.6% received insulin alone, and 26.8% received no diabetes medications.

**Table 1:** Characteristics of SHIELD respondents with and without type 2 diabetes mellitus who completed the 2004 and 2009 EQ-5D questionnaires, n = 6,284

**Characteristics**	**Type 2 diabetes mellitus (n = 1,741)**	**No diabetes (n = 4,543)**	**p-value**
Age, years, mean (SD)	60.6 (11.7)	56.1 (15.0)	<0.001
Women,%	60.2	62.4	0.25
White,%	85.4	89.5	<0.001
Education, high school degree or less,%	35.1	28.5	<0.001
Household income, <$40,000,%	51.9	41.8	<0.001
Body mass index, kg/m^2^, mean (SD)	33.7 (8.0)	29.8 (6.9)	<0.001
Duration of diabetes, years, mean (SD)	9.0 (7.8)	Not applicable	

Cross-sectionally, mean EQ-5D scores for 2004 and 2009 were significantly lower among T2DM respondents compared with respondents without diabetes (p < 0.0001 for both index and VAS scores) (Table 2). Longitudinally, there was a significantly greater decline in EQ-5D index score in the T2DM group (mean decline −0.031) compared with respondents without diabetes (−0.016) over the 5-year period (p = 0.001) (Table 2). EQ-5D VAS score declined over 5 years for both groups, with a change of −1.4 for T2DM respondents and −0.6 for respondents without diabetes. The difference between the T2DM and No diabetes groups in change in EQ-5D VAS scores was not statistically significant (p = 0.09).

**Table 2:** Mean scores and mean change in scores for EQ-5D among SHIELD respondents with and without type 2 diabetes mellitus

**EQ-5D**	**Type 2 diabetes mellitus (n = 1,741)**	**No diabetes (n = 4,543)**	**P-value**
2004 scores, mean (SD)			
Index	0.798 (0.174)	0.838 (0.161)	<0.0001
Visual analog	69.05 (19.83)	76.63 (18.43)	<0.0001
2009 scores, mean (SD)			
Index	0.767 (0.186)	0.822 (0.164)	<0.0001
Visual analog	67.63 (19.62)	76.00 (18.04)	<0.0001
Change from 2004 to 2009, mean (SD)			
Index	−0.031 (0.158)	−0.016 (0.141)	0.001
Visual analog	−1.416 (18.112)	−0.632 (15.821)	0.09

Multivariate regression adjusted for demographic variables, including age, gender, race, education, income, and BMI. Compared with respondents without diabetes, T2DM respondents had a larger reduction in EQ-5D index score, after controlling for demographics (p = 0.001) (Table 3). After controlling for demographic variables, change in EQ-5D VAS score did not differ between T2DM respondents and respondents without diabetes (p = 0.12). Age and gender were significantly associated with change in EQ-5D score for both the index and VAS, after controlling for the other covariates.

**Table 3:** Multivariate linear regression for change in EQ-5D scores among adults with and without type 2 diabetes

**Variables**	**EQ-5D index score**		**EQ-5D visual analog score**	
	**Beta coefficient (SE)**	**p-value**	**Beta coefficient (SE)**	**p-value**
T2DM vs. No diabetes	−0.015 (0.004)	0.001	−0.762 (0.495)	0.12
Age (per 1 year)	−0.001 (0.000)	<0.0001	−0.053 (0.015)	<0.0001
Women vs. men	0.008 (0.004)	0.04	1.078 (0.441)	0.015
Black vs. white	0.004 (0.007)	0.57	−0.556 (0.836)	0.51
Other race vs. white	0.018 (0.013)	0.16	−2.481 (1.467)	0.09
Body mass index (per 1 kg/m^2^)	0.000 (0.000)	0.08	0.087 (0.030)	0.004
Income (referent: <$22,500)				
$22,500 − $39,999	−0.001 (0.006)	0.82	0.497 (0.641)	0.44
$40,000 − $59,999	−0.006 (0.006)	0.30	0.026 (0.676)	0.97
$60,000 − $89,999	0.002 (0.006)	0.70	−0.687 (0.691)	0.32
>$89,999	0.005 (0.006)	0.40	0.357 (0.709)	0.61
Education (referent: high school degree)				
Some high school	0.004 (0.010)	0.72	0.208 (1.137)	0.85
Some college	0.000 (0.005)	0.93	0.153 (0.553)	0.78
College graduate	0.002 (0.006)	0.71	0.512 (0.653)	0.43
Graduate courses/ degree	−0.001 (0.006)	0.88	0.170 (0.730)	0.82

Approximately 9.8% of T2DM respondents reported a diagnosis of retinopathy, 25.0% reported a diagnosis of neuropathy, and 3.3% reported a diagnosis of nephropathy. The decline in EQ-5D index score was significantly greater among T2DM respondents who reported retinopathy, compared with T2DM respondents without retinopathy (p = 0.017) (Table 4). Similarly, the decline in EQ-5D index score was significantly greater among T2DM respondents who reported prevalent neuropathy, compared with T2DM respondents without neuropathy (p < 0.0001). T2DM respondents with reported nephropathy had a decline in EQ-5D index score that was not significantly different from the decline among T2DM respondents without nephropathy (p = 0.43). For change in EQ-5D VAS score, T2DM respondents who reported retinopathy had a similar decline as T2DM respondents without retinopathy (p = 0.88). Although T2DM respondents who reported neuropathy had twice the decline in EQ-5D VAS score as T2DM respondents without neuropathy, the difference was not statistically significant (p = 0.43). Moreover, T2DM respondents who reported nephropathy had a significantly greater decline in EQ-5D VAS score compared with T2DM respondents without nephropathy (p = 0.01).

**Table 4:** Mean change in EQ-5D scores over 5 years among adults with type 2 diabetes mellitus with and without diabetic complications

	**Change in EQ-5D index score**		**Change in EQ-5D Visual analog score**	
	**Mean (SD)**	**p-value**	**Mean (SD)**	**p-value**
Retinopathy				
Yes (n = 171)	−0.058 (0.162)	0.017	−1.614 (20.66)	0.88
No (n = 1,570)	−0.028 (0.158)		−1.394 (17.82)	
Neuropathy				
Yes (n = 436)	−0.061 (0.174)	<0.0001	−2.005 (21.12)	0.43
No (n = 1,305)	−0.020 (0.152)		−1.219 (16.99)	
Nephropathy				
Yes (n = 58)	−0.047 (0.164)	0.43	−7.448 (20.61)	0.01
No (n = 1,683)	−0.030 (0.158)		−1.208 (17.99)	

## Discussion

Over a 5-year period in the present study, health status of respondents with T2DM declined significantly, compared with adults with no diabetes, indicating that the burden of disease has a long-term detrimental impact on the quality of life of individuals living with T2DM. The decline in EQ-5D index score over 5 years among T2DM respondents was twice the decline among respondents without diabetes (p = 0.001), and the decline in VAS score was 2.2 times greater, albeit not statistically significant. The greater decline in health status among T2DM respondents remained even after adjusting for baseline differences between groups. The absence of significantly greater decline in VAS score may be due to the VAS being a single question on overall health currently. As a generic measure of health status, the VAS may be more influenced by other major health events such as myocardial infarction, stroke, and depression than the index score. The significantly greater decline in EQ-5D index score in the T2DM group compared with respondents without diabetes is likely to impact utility scores for economic evaluations. QALYs are used as indicators of effectiveness or health outcome in economic evaluations [24]. Cost-effectiveness studies are required to help clinicians and healthcare decision-makers in determining the impact of antidiabetic medications on both patient outcomes and societal costs. Understanding patient preferences (index score) for health outcomes is important for economic evaluations of new therapies, as well as for understanding patient behavior and adherence to diabetes therapy regimens. Thus in the present study, adults with T2DM would have fewer QALYs gained compared with adults without diabetes.

The greater decline in EQ-5D index score over 5 years for T2DM respondents with reported retinopathy and neuropathy is likely to impact utility scores in economic evaluations among adults with T2DM. A mailed questionnaire to individuals with T2DM in Norway found that the EQ-5D index score was 0.85 for those without diabetic complications and 0.73 for those with complications [25]. A clinical trial of intensive blood glucose control versus standard treatment in China showed that patients with microvascular complications reported significantly lower EQ-5D index scores than those without complications; however, there was no significant difference in HRQOL after 5 years in both groups [26]. In the present study, the decline in EQ-5D scores was greater among T2DM respondents with diabetic complications. From these findings, the presence of diabetic complications should be accounted for in the evaluation of the economic burden of T2DM.

The baseline EQ-5D scores in the present study were similar to those in other studies. Grandy and colleagues [9] reported cross-sectional EQ-5D scores for the T2DM respondents from SHIELD in 2004 (0.78 for index and 66.8 for VAS), which were similar to those in the present study of T2DM respondents who completed both the 2004 and 2009 SHIELD surveys (index = 0.80, VAS = 69.0 in 2004). The EQ-5D has been widely used in studies assessing HRQOL in diabetes, in particular, clinical trials evaluating different diabetes treatments or disease management programs [14,26-30]. A multi-country European study found significantly lower EQ-5D VAS scores for T2DM patients who reported hypoglycemic symptoms compared with patients not reporting symptoms [28]. A simulation model of diabetes disease progression showed that healthier patients with T2DM enjoy more life years, QALYs, and more life years free of complications [31]. Recent studies have demonstrated the construct, convergent and discriminant validity, test-retest reliability, and responsiveness of the EQ-5D in T2DM [32]. In a review of studies in T2DM that used the EQ-5D, EQ-5D index scores ranged from 0.20 for severe diabetic peripheral neuropathic pain to 0.88 for the general population with good HbA1c levels [32]. Pooled mean EQ-5D index scores were calculated for 6 subgroups: general population 0.67 (95% CI: 0.59−0.75), no complications 0.76 (0.68−0.83), microvascular complications 0.73 (0.57−0.89), macrovascular complications 0.73 (0.57−0.88), diabetic peripheral neuropathic pain 0.45 (0.39−0.51), and retinopathy 0.57 (0.46−0.69) [32]. Together with the present study findings of a decline in ED-5D index score over 5 years, this would potentially indicate that the T2DM respondents declined in health and possibly had diabetic complications that would negatively impact their QALYs.

EQ-5D scores for the respondents without diabetes were high; index scores were 0.84 in 2004 and 0.82 in 2009. However, these scores were similar to adults in the US general population where the mean EQ-5D score was 0.871 for the total population, 0.884 for men and 0.860 for women, and 0.872 for whites and 0.854 for blacks [33]. Jia and Lubetkin [33] used the 2000-2003 Medical Expenditure Panel Survey data to determine that the mean EQ-5D score was 0.825 for US adults in the general population with less than a high school education and 0.859 for those with a high school degree; 28.5% of the respondents without diabetes in the present study had a high school degree or less. Thus, the EQ-5D scores in the present study are in alignment with the general US population.

The present study provides evidence of the impact of T2DM on HRQOL over 5 years in a large sample using a standardized, validated measure so that norm-based results are provided. However, the study has some limitations. The diagnosis of diabetes and diabetic complications were self-reported and could not be validated with laboratory tests or medical record reviews. However, this bias is similar between the groups compared in this study. Household panels, like the TNS NFO panel, tend to under-represent the very wealthy and very poor segments of the population and do not include military or institutionalized individuals. Glucose control, such as HbA1c level, was not collected in the SHIELD surveys, so its impact on the decline in HRQOL cannot be assessed in this study. It is possible that the detrimental impact of T2DM over the 5 years may in part be due to poor glucose control. The proportion of T2DM respondents receiving no diabetic medications increased over time from 19.2% to 26.8%. It is possible that some respondents had improved their glycemic control through diet and exercise thus lowering their need for anti-diabetic medications; however, SHIELD did not collect data to address this supposition.

Another factor limiting the interpretation of the data is that minimally important differences (MIDs) for the EQ-5D have not been identified for T2DM patients treated in clinical practice (rather than clinical trial populations). The MID of 0.07 for the EQ-5D index score has been suggested, although the MID was not derived within samples of patients with T2DM [34-36]. Neither the T2DM respondents nor respondents without diabetes met the MID criterion for the change in EQ-5D index score.

In conclusion, health status of respondents with T2DM declined significantly over a 5- year period compared with respondents with no diabetes, indicating that the burden of disease has a long-term detrimental impact on HRQOL for adults living with T2DM. The significantly greater decline in EQ-5D index score in the T2DM group compared with respondents without diabetes, as well as the decline among T2DM respondents with diabetic complications, is likely to impact utility scores (fewer QALYs) for economic evaluations.

## Abbreviations

BMI: Body mass index; EQ-5D: EuroQol – 5 dimensions; HRQOL: Health-related quality of life; MID: Minimally important difference; QALYs: Quality-adjusted life years; SHIELD: **S**tudy to **H**elp **I**mprove **E**arly evaluation and management of risk factors **L**eading to **D**iabetes; T2DM: Type 2 diabetes mellitus; TNS NFO: Taylor Nelson Sofres National Family Opinion; VAS: Visual analog scale.

## Competing interests

This research was funded by AstraZeneca LP. SG is an employee and stock holder of AstraZeneca LP. KMF received research funds from AstraZeneca LP.

## Authors’ contributions

SG provided substantial contribution to the conception and design of the study and interpretation of the data, reviewed and revised the manuscript critically for important intellectual content, and approved the final version of the manuscript. KMF provided substantial contribution to the design of the analysis, oversaw the analysis, provided the data interpretation, wrote the manuscript, and approved the final version of the manuscript.

## References

[B1] Mokdad AH Bowman BA Ford ES Vinicor F Marks JS Koplan JP The continuing epidemics of obesity and diabetes in the United States JAMA 2001 286 1195 1200 10.1001/jama.286.10.1195 11559264

[B2] American Diabetes Association Diabetes statisticshttp://www.diabetes.org/diabetes-basics/diabetes-statistics.

[B3] International Diabetes Federation Diabetes Atlas 5 http://www.diabetesatlas.org.

[B4] Center for Disease Control Leading causes of deathhttp//www.cdc.gov/nchs/fastats/lcod.htm.

[B5] Luscombe FA Health-related quality of life measurement in type 2 diabetes Value Health 2000 3 Suppl 1 15 28 16464206 10.1046/j.1524-4733.2000.36032.x

[B6] Grandy S Chapman RH Fox KM for the SHIELD Study Group Quality of life and depression of people living with type 2 diabetes mellitus and those at low and high risk for type 2 diabetes: findings from the Study to Help Improve Early evaluation and management of risk factors Leading to Diabetes (SHIELD) Int J Clin Pract 2008 62 562 568 10.1111/j.1742-1241.2008.01703.x 18266708 PMC2423273

[B7] Watkins K Connell CM Measurement of health-related QOL in diabetes mellitus Pharmacoeconomics 2004 22 1109 1126 10.2165/00019053-200422170-00002 15612830

[B8] Anderson RM Fitzgerald JT Wisdom K Davis WK Hiss RG A comparison of global versus disease-specific quality-of-life measures in patients with NIDDM Diabetes Care 1997 20 299 305 10.2337/diacare.20.3.299 9051376

[B9] Grandy S Fox KM EQ-5D visual analog scale and utility index values in individuals with diabetes and at risk for diabetes: findings from the Study to Help Improve Early evaluation and management of risk factors Leading to Diabetes (SHIELD) Health Qual Life Outcomes 2008 6 18 10.1186/1477-7525-6-18 18304340 PMC2266905

[B10] Weinstein MC Siegel JE Gold MR Kamlet MS Russell LB Recommendations of the panel on cost-effectiveness in health and medicine JAMA 1996 276 1253 1258 10.1001/jama.1996.03540150055031 8849754

[B11] De Boer AG Stalmeier PF Sprangers MA de Haes JC van Sandick JW Hulscher JB van Lanschot JJ Transhiatal vs extended transthoracic resection in oesophageal carcinoma: patients’ utilities and treatment preferences Br J Cancer 2002 86 851 857 10.1038/sj.bjc.6600203 11953814 PMC2364156

[B12] Tosteson AN Hammond CS Quality-of-life assessment in osteoporosis: health-status and preference-based measures Pharmacoeconomics 2002 20 289 303 10.2165/00019053-200220050-00001 11994039

[B13] Smith DS Krygiel J Nease RF Jr Sumner W 2nd Catalona WJ Patient preferences for outcomes associated with surgical management of prostate cancer J Urol 2002 167 2117 2122 10.1016/S0022-5347(05)65099-9 11956454

[B14] Rubin RR Peyrot M Chen X Frias JP Patient-reported outcomes from a 16-week open-label, multicenter study of insulin pump therapy in patients with type 2 diabetes mellitus Diab Technol Ther 2010 12 901 906 10.1089/dia.2010.0075 20879963

[B15] Van Nispen RMA de Boer MR Hoeijmakers JGJ Ringens PJ van Rens GHMB Co-morbidity and visual acuity are risk factors for health-related quality of life decline: five-month follow-up EQ-5D data of visually impaired older patients Health Qual Life Outc 2009 7 18 10.1186/1477-7525-7-18 PMC2654874 19243624

[B16] Loftus JV Sultan MB Pleil AM Macugen 1013 Study Group Changes in vision- and health-related quality of life in patients with diabetic macular edema treated with pegaptanib sodium or sham Invest Ophthalmol Vis Sci 2011 52 7498 7505 10.1167/iovs.11-7613 21896838

[B17] Bays HE Chapman RH Grandy S for the SHIELD Investigators’ Group The relationship of body mass index to diabetes mellitus, hypertension and dyslipidaemia: comparison of data from two national surveys Int J Clin Pract 2007 61 737 747 10.1111/j.1742-1241.2007.01336.x 17493087 PMC1890993

[B18] Bays HE Bazata DD Clark NG Gavin JR III Green AJ Lewis SJ Reed ML Stewart W Chapman RH Fox KM Grandy S Prevalence of self-reported diagnosis of diabetes mellitus and associated risk factors in a national survey in the US population: SHIELD (Study to Help Improve Early evaluation and management of risk factors Leading to Diabetes) BMC Public Health 2007 7 277 10.1186/1471-2458-7-277 17915014 PMC2222165

[B19] US Census Bureau Annual supplement to the current population survey: census bureau resident population estimates of the United States 2003 Washington, DC: US Census Bureau

[B20] Rabin R de Charro F EQ-5D: a measure of health status from the EuroQol Group Ann Med 2001 33 337 343 10.3109/07853890109002087 11491192

[B21] Group EQ EuroQol – a new facility for the measurement of health-related quality of life Health Policy 1990 16 199 208 10109801 10.1016/0168-8510(90)90421-9

[B22] EuroQol Group EQ-5D nomenclature 2012 http://www.euroqol.org/eq-5d/what-is-eq-5d/eq-5d/nomenclature.html.

[B23] Shaw JW Johnson JA Coons SJ US valuation of the EQ-5D health states: development and testing of the DI valuation model Med Care 2005 43 203 220 10.1097/00005650-200503000-00003 15725977

[B24] Torrance GW Feeny D Utilities and quality-adjusted life years Int J Technol Assess Health Care 1989 5 559 575 10.1017/S0266462300008461 2634630

[B25] Solli O Stavem K Kristiansen IS Health-related quality of life in diabetes: the association of complications with EQ-5D scores Health Qual Life Outcomes 2010 8 18 10.1186/1477-7525-8-18 20132542 PMC2829531

[B26] Zhang Q Zhang N Hu HL He Y Chen MW Wang XY Yang MG Li J Effect of intensive blood glucose control on quality of life in elderly patients with type2 diabetes in Anhui Province Chin Med J 2011 124 1616 1622 21740765

[B27] Clarke PM Hayes AJ Glasziou PG Scott R Simes J Keech AC Using the EQ-5D index score as a predictor of outcomes in patients with type 2 diabetes Med Care 2009 47 61 68 10.1097/MLR.0b013e3181844855 19106732

[B28] Alvarez-Guisasola F Yin DD Nocea G Qiu Y Mavros P Association of hypoglycemic symptoms with patients’ rating of their health-related quality of life state: a cross sectional study Health Qual Life Outcomes 2010 8 86 10.1186/1477-7525-8-86 20723229 PMC2936440

[B29] Quah JHM Luo N Ng WY How CH Tay EG Health-related quality of life is associated with diabetic complications, but not with short-term diabetic control in primary care Ann Acad Med Singapore 2011 40 276 286 21779616

[B30] Ose D Miksch A Urban E Natanzon I Szecsenyi J Kunz CU Freund T Health related quality of life and comorbidity. A descriptive analysis comparing EQ-5D dimensions of patients in the German disease management program for type 2 diabetes and patients in routine care BMC, Health Services Res 2011 11 179 10.1186/1472-6963-11-179 PMC3161848 21810241

[B31] Hayes AJ Clarke PM Voysey M Keech A Simulation of quality-adjusted survival in chronic diseases: an application in type 2 diabetes Med Decis Making 2011 31 559 570 10.1177/0272989X11409049 21636740

[B32] Janssen MF Lubetkin EI Sekhobo JP Pickard AS The use of the EQ-5D preference-based health status measure in adults with type 2 diabetes mellitus Diabet Med 2011 28 395 413 10.1111/j.1464-5491.2010.03136.x 21392061

[B33] Jia H Lubetkin EI The statewide burden of obesity, smoking, low income and chronic diseases in the United States J Public Health 2009 31 496 505 10.1093/pubmed/fdp012 19251766

[B34] Walters SJ Brazier JE Comparison of the minimally important difference for two health state utility measures: EQ-5D and SF-6D Qual Life Res 2005 14 1523 1532 10.1007/s11136-004-7713-0 16110932

[B35] Brazier J Roberts J Tsuchiya A Busschbach J A comparison of the EQ-5D and SF-6D across seven patient groups Health Econ 2004 13 873 884 10.1002/hec.866 15362179

[B36] Pickard AS Neary MP Cella D Estimation of minimally important differences in EQ-5D utility and VAS scores in cancer Health Qual Life Outcomes 2007 5 70 10.1186/1477-7525-5-70 18154669 PMC2248572

